# Automating Case Reporting of Chlamydia and Gonorrhea to Public Health Authorities in Illinois Clinics: Implementation and Evaluation of Findings

**DOI:** 10.2196/38868

**Published:** 2023-03-14

**Authors:** Ninad Mishra, Reynaldo Grant, Megan Toth Patel, Siva Guntupalli, Andrew Hamilton, Jeremy Carr, Elizabeth McKnight, Wendy Wise, David deRoode, Jim Jellison, Natalie Viator Collins, Alejandro Pérez, Saugat Karki

**Affiliations:** 1 Division of STD Prevention Centers for Disease Control and Prevention Atlanta, GA United States; 2 Division of Infectious Diseases Office of Health Protection Illinois Department of Public Health Springfield, IL United States; 3 AllianceChicago Chicago, IL United States; 4 Lantana Consulting Group East Thetford, VT United States; 5 Public Health Informatics Institute Atlanta, GA United States

**Keywords:** public health surveillance, sexually transmitted diseases, gonorrhoea, chlamydia, electronic case reporting, eCR, health information interoperability, electronic health records, EHR, case reporting, automated, reporting, recording, patient records, cases, health care system, semantic, interoperability, implementation

## Abstract

**Background:**

Chlamydia and gonorrhea cases continue to rise in Illinois, increasing by 16.4% and 70.9% in 2019, respectively, compared with 2015. Providers are required to report both chlamydia and gonorrhea, as mandated by public health laws. Manual reporting remains a huge burden; 90%-93% of cases were reported to Illinois Department of Public Health (IDPH) via electronic laboratory reporting (ELR), and the remaining were reported through web-based data entry platforms, faxes, and phone calls. However, cases reported via ELRs only contain information available to a laboratory facility and do not contain additional data needed for public health. Such data are typically found in an electronic health record (EHR). Electronic case reports (eCRs) were developed and automated the generation of case reports from EHRs to be reported to public health agencies.

**Objective:**

Prior studies consolidated *trigger* criteria for eCRs, and compared with manual reporting, found it to be more complete. The goal of this project is to pilot standards-based eCR for chlamydia and gonorrhea. We evaluated the throughput, completeness, and timeliness of eCR compared to ELR, as well as the implementation experience at a large health center–controlled network in Illinois.

**Methods:**

For this study, we selected 8 clinics located on the north, west, and south sides of Chicago to implement the eCRs; these cases were reported to IDPH. The study period was 52 days. The centralized EHR used by these clinics leveraged 2 of the 3 case detection scenarios, which were previously defined as the *trigger*, to generate an eCR. These messages were successfully transmitted via Health Level 7 electronic initial case report standard. Upon receipt by IDPH, these eCRs were parsed and housed in a staging database.

**Results:**

During the study period, 183 eCRs representing 135 unique patients were received by IDPH. eCR reported 95% (n=113 cases) of all the chlamydia cases and 97% (n=70 cases) of all the gonorrhea cases reported from the participating clinical sites. eCR found an additional 14 (19%) cases of gonorrhea that were not reported via ELR. However, ELR reported an additional 6 cases of chlamydia and 2 cases of gonorrhea, which were not reported via eCR. ELR reported 100% of chlamydia cases but only 81% of gonorrhea cases. While key elements such as patient and provider names were complete in both eCR and ELR, eCR was found to report additional clinical data, including history of present illness, reason for visit, symptoms, diagnosis, and medications.

**Conclusions:**

eCR successfully identified and created automated reports for chlamydia and gonorrhea cases in the implementing clinics in Illinois. eCR demonstrated a more complete case report and represents a promising future of reducing provider burden for reporting cases while achieving greater semantic interoperability between health care systems and public health.

## Introduction

### Background

#### Burden of Sexually Transmitted Infections in Illinois

Based on 2019 surveillance data, the disease burden for chlamydia and gonorrhea has increased by 16.4% and 70.9%, respectively, in Illinois since 2015 [1]. Public health laws mandate that these sexually transmitted infections be reported to Illinois Department of Public Health (IDPH) by health care providers and clinical laboratories within 7 business days [2]. Health care providers in Illinois generally report sexually transmitted infection cases via a web-based data entry platform; health systems and laboratories generally report their case results by electronic laboratory reporting (ELR). For many health care providers, reporting via the web-based platform poses an enormous workforce burden, and many report barriers to reporting such as lack of capacity, difficulty extracting relevant information, confusing vocabulary standards, etc [3]. Consequently, case reporting via electronic health records (EHRs) is a welcome prospect for all parties—reporting entities and public health agencies.

#### Public Health Surveillance and Informatics

Public health surveillance as a distinct discipline of public health was described by Langmuir [4] with the emphasis of ongoing and systemic collection of relevant data, consolidation, and analysis, followed by regular dissemination to all who need to know and can take public health action [5]. The role of public health informatics in facilitating surveillance is: “1) to improve timelines and completeness of data collection and analysis and 2) to free human resources to focus on the areas that require the most creative thought and to do the work that technology cannot” [6]. The Centers for Disease Control and Prevention (CDC) published the agency’s vision for public health surveillance in the 21st century [7] and recognized an opportunity to improve data quality and timeliness by accessing EHRs [8].

ELR and electronic case reporting (eCR) are creating a paradigm shift in public health surveillance. ELR is the automated messaging of laboratory reports of notifiable cases and has been widely adopted in the United States [9]. eCR is the automated generation of case reports from EHRs and subsequent reporting to public health agencies [10,11]. Both eCR and ELR were found to be more complete and timelier than paper-based reporting [12,13]. These approaches represent an advancement toward better semantic interoperability to support public health surveillance [14], greatly reducing the burden of reporting from clinical providers and improving the completeness and timeliness of those reports [15]. However, further evaluation and consideration is required to achieve a greater level of success and widespread implementation.

### Prior Work in eCR Architecture for Chlamydia and Gonorrhea

The Council of State and Territorial Epidemiologists determines the case definition of notifiable conditions and maintains position statements for these conditions, including for chlamydia and gonorrhea [16-18]. An earlier study by Mishra et al [12] leveraged these position statements to develop the case detection logic for chlamydia and gonorrhea in EHRs. As an outcome of this study, value sets were created using national health care data standards (eg, International Classification of Diseases, Tenth Revision, Clinical Modifications; Logical Observation Identifiers Names and Codes [LOINC]; and Systematized Nomenclature of Medicine – Clinical Terms [SNOMED-CT]) [19] and Health Level 7 (HL7) [20]. Mishra et al [12] also compared eCR reporting with manual reporting and found that eCR increased provider reporting and improved the completeness of those case reports [12]. This study further consolidated the *trigger* for eCRs (when the case-detection logic is met in the EHR) based on the following three scenarios: (1) when an individual is diagnosed, (2) when a confirmatory laboratory result (named organism) is returned, or (3) a combination of laboratory test performed and result indicating the presence of infection is found (without the organism named in the result) [21]. Subsequently, this eCR architecture was implemented in Oregon simultaneously to our implementation in Illinois. In both the Oregon implementation and this study reporting the Illinois findings, the eCRs were generated via the HL7 electronic initial case report (eICR) standard [20,22]. The Oregon implementation found that this eCR architecture successfully reported cases of chlamydia and gonorrhea to public health while at the same time improving on the completeness compared to ELRs for the same cases [22].

### Objective

The primary goal of this project was to pilot standards-based eCR for chlamydia and gonorrhea in 2 participating jurisdictions (public health agencies of the states of Illinois and Oregon), study the completeness of data between ELRs and eCRs, and disseminate the findings to promote adoption. In this process, we not only furthered the previous work by Mishra et al [12] but also learned about local variations while implementing the same architecture of eCR in 2 separate state health departments [12,22]. We recognize that local codes in EHRs are very common, and mapping them is a challenge [14]. Due to the challenges posed by variations in nonstandard codes used in EHRs, every implementation of eCR becomes an exercise of local implementation with some degree of customization. We further evaluated this implementation experience at a large health center–controlled network in Illinois. Additionally, we evaluated the completeness of eCR and compared it to ELR.

## Methods

### Ethical Review and Study Duration

For this evaluation, case-related information was sent from the EHR to IDPH, the State of Illinois’s public health agency. An internal review committee of the participating clinics deemed that an Institutional Review Board review was not necessary for this project. The study period was a little over 7 weeks (or 52 days), which began on August 12, 2020, and ended on October 2, 2020.

### Implementation Partners and Clinical Setting

#### Clinical Site and EHR Platform

We sought a network of clinics that shared a common platform of centrally hosted EHR. This would allow for scaling up across multiple clinical sites using a single implementation of the eCR architecture. AllianceChicago is a health center–controlled network that supports 45 ambulatory primary care practices in 19 states. For this evaluation, we selected 8 clinics operated by Near North Health, a federally qualified health center, located on the north, west, and south sides of Chicago [23]. In 2019, the selected clinical sites served 37,223 patients for a total of 122,277 visits. These clinical sites used AthenaHealth as the EHR [24] and QVERA as the interface engine [25].

#### Public Health Department

IDPH has a centralized IT infrastructure that uses the Illinois National Electronic Disease Surveillance System (I-NEDSS), a home-grown person-based and event-based system [26]. Health care providers and clinical laboratories are required to report all cases of chlamydia and gonorrhea within 7 working days [2,27]. Prior to this project, IDPH accepted reports via ELR, electronic provider report (a custom flat file generated by the health care provider), provider reports via a web-based data entry platform, faxes, and in rare instances, phone calls. An internal review (unpublished) of IDPH's I-NEDSS surveillance system revealed that in 2020, 90%-93% of the chlamydia and gonorrhea cases originated from ELRs.

### Implementation of eCR Architecture and Method of Receiving Case Reports

#### Implementing eCR Architecture at Clinical Sites

**Figure 1:**
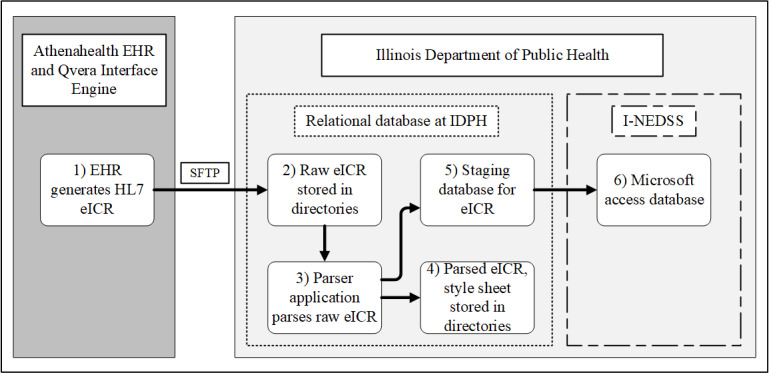
Workflow depicting electronic case report (eCR) generation, transportation, and ingestion. EHR: electronic health record; eICR: electronic initial case report; HL7: Health Level 7; I-NEDSS: Illinois National Electronic Disease Surveillance System; IDPH: Illinois Department of Public Health; SFTB: secure file transfer protocol.

The reference eCR implementations were defined in the previous study [12]. Some degree of customization was necessary to accommodate existing public health workflows, available code terminologies, and data parsing. The EHR leveraged 2 of the 3 case-detection scenarios [21]—scenario 1 (encounter diagnoses) and scenario 3 (name of the laboratory test performed and abnormal result flag), but not scenario 2 (name of the microorganism identified). While the case detection value sets include standard code concepts and terminologies, a given EHR instance may not have equivalent codes that reflect all 3 scenarios. This was the case for the Illinois implementation where the EHR in use contained codes aligned with Scenarios 1 and 3 only. The case detection logic is designed to work in the background of the EHR to detect chlamydia and gonorrhea cases using a predefined case detection algorithm consisted of value sets using industry standard terminologies (eg, International Classification of Diseases, Tenth Revision, Clinical Modifications; LOINC; and SNOMED-CT) [21], with no need for additional action on the part of the patient care team. Once the case detection logic was met, the EHR created XML documents in accordance with the HL7 eICR [28] and delivered them to IDPH via Secure File Transfer Protocol (Step 1, Figure 1).

#### Receiving and Ingesting eCR at Public Health Agency

Directly ingesting eCRs into I-NEDSS was deemed out of scope due to project timeline and the extent of invasive intervention required to make modifications in the surveillance system. The raw eCRs were, instead, stored in a directory at IDPH (Step 2, Figure 1) and parsed by XML Harvester [29], an open-source XML parser application (Step 3, Figure 1). The parser application was housed in a relational database and passed the reformatted records to the staging database (Step 5, Figure 1). Relevant tables were identified by mapping data elements to the I-NEDSS and ingested into a Microsoft Access database for analysis (Step 6, Figure 1).

### Data Collection and Analysis Methods

AllianceChicago tallied a count of eCRs generated daily throughout the pilot period and extracted data directly from the EHR. In some instances, manual inspection was performed on the captured data. Once the eCRs were received at IDPH, they were evaluated for the following: (1) compliance with the trigger logic for confirmed chlamydia and gonorrhea laboratory results or diagnosis, (2) completeness of key data elements, (3) timeliness of reporting, and (4) the degree to which eCR laboratory results matched ELR reporting during the study period. We compared the timeliness of eCR to ELR by calculating the median time from documented patient encounter to the time the public health agency received eCR and ELR for an individual.

## Results

### Case Report Throughput and Case Detection Metrics

#### eCR Throughput

During the study period, 11,192 encounters were logged in the EHR across all 8 clinical sites for various types of clinical care. During the study period, IDPH received 202 eCRs which were valid as per the HL7 requirements and stored in the staging database. Of these 202 eCRs, 19 (9.4%) did not meet the Council of State and Territorial Epidemiologists case definition and were not tallied. They were rejected because a manual review determined that they were not positive chlamydia or gonorrhea eCR tests or diagnosis. Therefore, a total of 183 eCRs representing 135 unique patients (some patients had both chlamydia and gonorrhea) were deemed valid and met the case definitions.

#### Case Detection Metrics

There were 113 instances where the case detection logic was satisfied for chlamydia; 80% (90/113) were based on scenario 3 (name of the laboratory test performed and abnormal result flag) and 20% (23/113) were based on both scenario 1 (encounter diagnoses) and scenario 3 (Table 1). Similarly, for the 70 instances where the case detection logic was satisfied for gonorrhea, 61% (43/70) were based on scenario 3, and 39% (27/70) were based on scenarios 1 and 3. Of note, there were no instances when scenario 1 (encounter diagnoses) alone was met, but instead, all of them had a corresponding confirmatory laboratory test (Table 1). eCR reported 95% (n=113 cases) of all the chlamydia cases that were reported from the participating clinical sites (Table 1). However, ELR reported 6 additional cases of chlamydia, which were not reported via eCR. eCR reported 97% (n=70 cases) of all the gonorrhea cases found by ELR, finding an additional 14 cases (19%) that were not reported via ELR (Table 1). ELR reported 2 additional cases of gonorrhea not detected by eCR. ELR reported 100% (119/119) of chlamydia cases but only 81% (58/72) of gonorrhea cases.

**Table 1:** Case detection metrics for electronic case reporting (eCR) and electronic laboratory reporting (ELR).

Variables					Chlamydia (n=119), n (%)							Gonorrhea (n=72), n (%)			
					Reported by ELR							Reported by ELR			
					Yes			No			Yes				No
**Reported by eCR**															
	**Yes**														
		Scenario 1 (encounter diagnoses) only	0			0			0				0		
		Scenario 3 (name of the laboratory test performed and abnormal result flag) only	90 (76)			0			33 (57)				10 (71)		
		Both scenarios 1 and 3	23 (19)			0			23 (40)				4 (29)		
	**No**														
		No eCR was generated	6 (5)			0			2 (3)				0		
Total, n					119			0			58				14

### eCR Data Completeness and Timeliness

Key elements for public health reporting such as patient and provider names were complete in all eCRs and ELRs (Table 2). Race and ethnicity were slightly lower in eCR (n=113, 95%) than ELR (n=119, 100%; Table 2). However, eCR was found to report additional clinical data such as social history information, which is important for portraying a more complete epidemiologic picture but was beyond the scope of this evaluation. History of present illness and reason for visit were both reported on 82% (97/119) of eCR in this study; symptoms, diagnosis, and medications were reported on 33% (39/119), 23% (27/119), and 22% (26/119) eCRs, respectively (Table 2). While pregnancy is supported in the HL7 eICR standard [21], total absence of pregnancy information is due to the configuration of the EHR and is specific to this implementation.

The median time from the documented patient encounter to the time IDPH received ELR was 4 days. Similarly, the median time for scenario 1 (encounter diagnosis) was 11 days, and for scenario 3 (laboratory result) was 6 days.

**Table 2:** Comparison of the completeness in electronic case reporting (eCR) and electronic laboratory reporting (ELR).

eCR data element	Complete, n (%)	ELR data element	Complete, n (%)
Provider name	119 (100)	Ordering provider	119 (100)
Provider phone	119 (100)	Oder callback phone number	119 (100)
Provider fax	0 (0)	N/A^a^	N/A
Provider email	90 (76)	N/A	N/A
Patient name	119 (100)	Patient name	119 (100)
Patient phone	119 (100)	Phone number (home); phone number (business)	119 (100)
Patient email	29 (24)	N/A	N/A
Street address	119 (100)	Patient address	119 (100)
Birth date	119 (100)	Date or time of birth	119 (100)
Patient sex	119 (100)	Administrative sex	119 (100)
Race	113 (95)	Race	119 (100)
Ethnicity	113 (95)	Ethnic group	119 (100)
Preferred language	119 (100)	N/A	N/A
Pregnant	0 (0)	N/A	N/A
History of present illness	97 (82)	N/A	N/A
Reason for visit	97 (82)	Reason for study	0 (0)
Date of onset	39 (33)	N/A	N/A
Symptoms (list)	39 (33)	N/A	N/A
Diagnoses	27 (23)	N/A	N/A
Date of diagnosis	28 (24)	Date or time of the analysis	119 (100)
Medication administered (list)	26 (22)	N/A	N/A

^a^N/A: not applicable.

## Discussion

### Principal Findings

We implemented an approach to case reporting that automated and provided more complete, timely, and relevant information to public health authorities for chlamydia and gonorrhea in Illinois. This approach furthered data exchange between health care providers and public health by using health care data standards (SNOMED-CT, LOINC) to meet case detection logic in the EHR [12,21] and messaging standards (HL7 eICR) as the transport mechanism [20]. This method could facilitate better public health surveillance and inform public health practice. While ELRs comprised 90% of all cases of notifiable conditions reported to IDPH in 2020, eCR can provide better clinical information than the ELR is capable of reporting. Additionally, we demonstrated that eCR can be configured to retrigger at a later date to capture information not available at the time the initial case definition was met.

In this study, ELR detected 6 additional chlamydia cases and 2 additional gonorrhea cases, and eCR detected 14 additional cases of gonorrhea. These 14 cases were detected via scenarios where laboratory results were present in the EHR. We did not investigate why these were missed by ELRs, but some hypotheses include as follows: (1) specimen was collected toward the end of the study period (median time for receipt of ELR by IDPH was 4 days), (2) laboratory where the test was done could have had problems leading to delayed reporting, (3) specimens could have been tested by out-of-jurisdiction labs, and (4) other unknown reasons. Similarly, eCR detection logic missed 6 chlamydia and 2 gonorrhea cases. Potential reasons include the following: (1) faulty local code mapping; (2) if patient was seen toward the end of the study period, eCR may not have triggered yet (could take up to 11 days for IDPH to receive eCR, compared to ELR, which took 4 days); and (3) other unknown reasons. Of note, among all the cases reported by the clinical sites, eCR reported 95% and 97% of all chlamydia and gonorrhea cases, respectively. We also found that eCR contains more complete information compared to ELR and allowed for the collection of additional data such as diagnoses, treatment, and other clinical information that are typically not available in ELR. However, these additional data elements were not available on all eCRs. Some records reflect clients who were tested or diagnosed at external locations whose records were subsequently scanned into the EHR system as PDF files with no accompanying lab records. More work needs to be done to explore whether these were not available at the time of the case report generation or if this information was present in some other unstructured format not represented as a standard vocabulary such as SNOMED-CT.

An earlier study by Mishra et al [12] had developed the case detection logic, curated the value sets needed for this eCR architecture, and collaborated with a clinical partner to demonstrate the case detection and eCR efficacy. Another study in Oregon, simultaneously conducted with this evaluation, also implemented this approach with clinical sites reporting to the Oregon Health Authority. They reported on the local customization required for that implementation and differed from this study in that the Oregon Health Authority had existing infrastructure to consume eCR via its interface engine into its proprietary surveillance system [21]. A broader adoption of eCR will be a step toward bridging the information gap between health systems and public health. Such steps toward improved semantic interoperability will allow a timely and better understanding of epidemiology, will inform the development of effective policy and most relevant updates to treatment guidelines, and will reduce burdens for both health systems and public health agencies by automating case reporting and investigating cases for more information.

### Limitations

The EHR had limited Fast Healthcare Interoperability Resources capability. The health centers had to collaborate with the EHR vendor to develop, test, and release a beta version of eCR that could be specifically used for this project. eCR standards allow for data elements that are above and beyond those that have been received through traditional reporting. This information may be present in an unstructured format without any semantic representation. An eCR triggered by laboratory results (scenario 3) would contain information in a structured format with semantic representation, while for scenario 1, diagnoses may not be available in a structured format but may be represented in an unstructured, narrative format, resulting in a lower number of triggers. Some additional examples of unstructured data elements include those found in the “Problems,” “History of present illness,” “Reason for visit,” “Social history,” and so on, and could explain why symptoms, diagnosis, and medications were reported only on 33%, 23%, and 22% of the eCRs, respectively.

I-NEDSS, the surveillance system at IDPH, required additional resources to consume eCR. Any addition to the surveillance system’s capability would require extensive modification. Due to the constraint of project timeline and lack of dedicated resources for this activity, we elected not to reconfigure the surveillance information system to receive eCR. Instead, we used a parser application and created a staging database that could be mapped to the data elements of the surveillance information system. This challenge of ingesting eCRs into surveillance information systems is likely to be far more common across health departments in the United States and needs additional work to create a standardized and scalable approach. We did not quantify the burden of manual reporting, and how much of that burden was potentially alleviated via automated case reporting via eCR. Future studies could provide insight into the resources saved via such automated reporting methods.

### Conclusions

The eCR approach to public health surveillance successfully identified and created automated case reports for chlamydia and gonorrhea cases in the selected Illinois clinics. This approach also demonstrated a more complete case report with additional demographic, clinical, and treatment information. eCR reduced the burden of reporting cases on clinical providers and represents a promising future of greater semantic interoperability between health care systems and public health by automating the case report using health care data standards in a scalable manner.

## Abbreviations

CDC: Centers for Disease Control and Prevention
eCR: electronic case reporting
EHR: electronic health record
eICR: electronic initial case report
ELR: electronic laboratory reporting
HL7: Health Level 7
I-NEDSS: Illinois National Electronic Disease Surveillance System
IDPH: Illinois Department of Public Health
LOINC: Logical Observation Identifiers Names and Codes
SNOMED-CT: Systematized Nomenclature of Medicine – Clinical Terms
